# “*It’s just like a blood transfusion”*: perceptions on the use of donated breast milk in selected hospitals in central Uganda: a qualitative study

**DOI:** 10.1186/s12889-023-15648-1

**Published:** 2023-05-16

**Authors:** Mary Gorreth Namuddu, David Mukunya, Victoria Nakibuuka, Esther Amulen, Ritah Nantale, Juliet Kiguli

**Affiliations:** 1 School of Public Health, Makerere University, College of Health Sciences, P.O. Box 7072, Kampala, Uganda; 2 Department of Community and Public Health, Busitema University, Faculty of Health Sciences, P.O. Box 1460, Mbale, Uganda; 3 Department of Research, Nikao Medical Center, Kampala, Uganda; 4 Department of Paediatrics, Nsambya Hospital, P.O. Box 7146, Kampala, Uganda

**Keywords:** Human milk banking, Perceptions, Donated breast milk

## Abstract

**Background:**

Breast milk is crucial for the nutritional and developmental milestones in the first two years of life. Uganda has recognized the need for a human milk bank as an opportunity that offers reliable and healthy milk to babies who lack access to their mothers. However, there is little information on the perceptions towards donated breast milk in Uganda. This study aimed to explore the perceptions of mothers, fathers, and health workers on the use of donated breast milk at Nsambya and Naguru hospitals in Kampala district, central Uganda.

**Methods:**

A qualitative descriptive study was conducted at Nsambya and Naguru hospitals in central Uganda. The study consisted of 8 focus group discussions (FGDs) of 6 participants each and 19 key informant interviews (KIIs) among mothers, fathers, and health workers. Participants were purposively selected. Data collected were transcribed, translated from Luganda to English, and analyzed using thematic analysis. All data were organized and managed in Nvivo version 12.0.

**Results:**

A total of 67 participants were involved in the study. Two main themes were identified: positive perceptions and negative perceptions. Participants linked donated breast milk to blood transfusion, believed it had nutrients comparable to the biological mother’s milk, and thought it was an opportunity to avoid formula or cow milk and help babies that cannot access breast milk. However, the notable negative perceptions were; the feeling that donated breast milk is disgusting, could result in acquiring non-parental genes and traits, and that it was unsafe. Participants also feared that donated breast milk could be expensive and affect the bond between mother and child.

**Conclusion:**

In summary, participants had positive perceptions about donated breast milk but were concerned about the potential side effects. Health workers should take extra precautions to ensure that donated breast milk is safe. The development of appropriate information and communication programs to sensitize the public about the benefits of donated breast milk will improve the uptake. Further research should focus on understanding the social-cultural beliefs regarding donated breast milk.

## Background

Insufficient breast milk has been observed among women who deliver by cesarean section, preterm births and those with maternal illnesses. Due to these conditions, the onset of lactation occurs later than 72 h after birth [1–5]. Lack of access to breastmilk has contributed to the failure to meet the recommended breastfeeding standards. Worldwide, approximately 40% of newborns in Neonatal Intensive Care Units (NICU) do not get breast milk from their mothers until days or weeks later [6]. In low- and middle-income countries, only 45.7% of all babies under 6 months are exclusively breastfed and 51.9% are initiated on breast milk in the first hour of birth [7]. Although Uganda has achieved exclusive breastfeeding rates above the global target of 50% by 2025 [8, 9], the adherence to exclusive breast feeding decreases sharply with age from 83 to 69% and 43% between 0 and 1, 2–3 and 4–5 months respectively [10]. In addition, nearly 34% of babies in Uganda do not initiate breastfeeding in the first hour of birth [10].

In situations where mother’s own milk is inaccessible, World Health Organization (WHO) recommends donated breast milk as the next preferred alternative [11–13]. Donated breast milk is the milk expressed by a lactating mother and then processed by a human milk bank for use by a recipient other than the biological child [14]. Despite the recommendations and benefits of breast milk, Uganda, like other low-income countries with high neonatal mortality, lag in establishing and advancing human milk banks [14, 15]. In Uganda, about 42% of children under five years die from preventable causes, including failure to breastfeed [16]. Breastfeeding is believed to be the best preventive intervention for child mortality [9, 17].

Currently, the need for a human milk bank has risen to the forefront of public health agendas in Uganda. St. Francis hospital Nsambya located on the outskirts of Kampala city, officially launched its first bank in November 2021. It intends to provide pasteurized breast milk to preterm and other babies whose mothers lack or have inadequate breast milk. However, there is little information on how the would-be users perceive the use of donated breast milk for infant feeding. Mothers use donated breast milk for various reasons, including awareness and knowledge of human milk banking, religious and cultural beliefs, ethical issues, attitudes, safety concerns, failure to breastfeed, and so forth [18–20]. Preceding the adoption of a new sensitive health intervention, especially in communities with cultural and other contextual differences coupled with a high prevalence of infectious diseases such as HIV, an in-depth consideration of perceptions and acceptability of the concept by the potential recipients is very important [21]. In addition, initiatives that target already vulnerable populations should be tested for acceptability to ensure that they are only for their benefit. For this study, perception refers to how the participants understand or interpret or feel about donated breast milk based on their thoughts, senses, and experiences. The study findings will help create awareness, thus providing a framework for lobbying and future training programs on the intervention. Therefore, support the implementation and sustainability of human milk banks in Uganda. The study will also help to inform human milk banking policies and guidelines in Uganda. This study sought to explore the perceptions of mothers, fathers and health workers on the use of donated breast milk at Nsambya and Naguru hospitals in central Uganda.

## Methods

### Study design

A cross-sectional study that employed a qualitative descriptive design was conducted. This approach allows the study of subjective things, like experiences and perceptions [22–24]. Qualitative descriptive research aligns with theories that use interpretative and naturalistic approaches [22, 24]. These philosophical perspectives agree that reality can be in various dynamic contexts and perceived differently depending on the subject [24]. The research design provided an excellent method to explore participants’ perceptions towards donated breast milk by allowing their true reflection of the subject at hand.

### Study setting, participants and sampling procedures

The study was carried out at St. Francis hospital Nsambya and China-Uganda Friendship hospital (Naguru hospital) in Kampala, the capital of Uganda, from July to October 2020. The hospitals were selected using purposive sampling. Naguru hospital is one of the referral public hospitals, with a substantial number of premature births (1,095 births between June 2018 and May 2019) [25]. For this reason and accessibility, it was chosen for the study. Nsambya hospital is a private hospital where the first human milk bank is established. Exploring perceptions towards donated breast milk in both private and public hospitals enabled the representation of views of different categories of potential users.

The study participants involved fathers, health workers (midwives, nurses, and paediatricians), and mothers (pregnant and lactating mothers, grandmothers, Mothers ever used donated breast milk, and mothers of preterm babies).

Pregnant mothers attending the antenatal unit and lactating mothers in the immunization unit between July and October 2020 were enrolled. Also, mothers that had used donated breast milk before the study and mothers of preterm babies receiving care at the time of data collection in selected hospitals were included. Grandmothers and fathers who were caring for sick relatives and health workers at the selected hospitals were also included in the study. Study participants were purposively selected either through patient records or because they were attending or working at the selected hospitals. All study participants were recruited one-on-one by the principal researcher and research assistants. The hospital staff (nurses) supported the study recruitment by identifying mothers who had premature babies or had ever used donated breast milk from the patient records on the day of data collection. Only participants that gave consent were included in the study. All pregnant mothers who were critically ill and lactating mothers with unhealthy babies were excluded. All health workers are crucial for the study’s purpose. However, given the health worker-patient ratio in the hospitals, conducting qualitative interviews with all of them was impossible due to workload and task prioritization. Therefore, we excluded health workers with limited interaction with the mothers. Health workers were selected based on their professional expertise and experience in caring for vulnerable babies.

#### Data collection

Four focus group discussions (FGDs) were held in each of the two selected hospitals in groups of six participants. The FGDs conducted per hospital included; 1 group with pregnant mothers, 1 with fathers,1 with grandmothers, and 1 with lactating mothers leading to a total of 8 FGDs for the study. The variation of the participants was based on demographic composition such as; age, number of children, and education level. In addition, 19 Key Informant Interviews (KIIs) were conducted. KIIs were carried out in private rooms at the selected hospitals to avoid distractions, facilitate clear recordings and ensure that the responses were anonymous. KIIs involved 2 midwives, 2 nurses and 1 paediatrician from each hospital. Six KIIs were conducted with mothers of preterm babies, of which 4 were from Naguru hospital and 2 from Nsambya hospital. All three KIIs conducted among mothers who had ever used donated breast milk were from Nsambya hospital. We could not find any mother from Naguru hospital that had used donated breast milk, as we had planned. The study involved a total of 67 participants.

The research team explained the study’s purpose to the participants, obtained their consent, and collected demographic information (Table 1) before starting any discussion or interview. We assigned participants random numbers as codes before collecting data to prevent them from being associated with the study findings. All FGDs and some of the KIIs with the mothers were conducted in Luganda, the most spoken local language. All interviews with health workers were conducted in English. The research team understood the languages used in the study. The research team involved a principal researcher with a master’s in Public Health Nutrition and four research assistants with degrees. In all FGDs, there was a facilitator who was the principal researcher, a note-taker, and an assistant moderator who also helped to keep track of the audio recordings. KIIs involved a facilitator and one note-taker. FGD and KII guides with open-ended questions were developed after the literature review to guide data collection. We asked questions while probing to obtain all the information and clarify what the participants did not understand. The guides had questions related to participants’ demographic information, whether participants had heard of donated milk and human milk banking, current feeding practices and options for infants without access to mother’s milk, and perceptions toward donated breast milk use. The tools were first translated from English to Luganda, reviewed for face validity, and adjusted accordingly before carrying out the study. The research assistants were trained on the content of the tools, study objectives, and how to facilitate group discussions and conduct interviews. The interviews and discussions lasted for about 30 to 60 min. Detailed hand-written notes were taken by the note-takers, and audio recorded the interviews and discussions with permission from participants. The number of FGDs and KIIs conducted was determined by the principle of saturation. Data analysis started while we were in the field. We analyzed the data as we went along until we did not find any new emergent themes.

#### Data management and analysis

Data obtained were transcribed verbatim by the principal researcher, translated from Luganda to English for the KIIs and FGDs conducted in Luganda to ensure accuracy, and saved in word files. Recordings were played and listened to multiple times to ensure that transcription was accurate. Also compared audio transcripts to the detailed notes taken during the discussions and interviews for accuracy. For the reliability of the study findings, we triangulated data from KIIs and FGDs. Thematic analysis was used to analyze the data.

We used a deductive approach to analyze data by focusing on research questions. Each segment of the data that was relevant to the research question was coded. We used guidelines set out by Braun and Clarke to analyze the data [26]. We read the transcripts repeatedly, organized the data and generated initial codes. We did not have specific codes beforehand hence used open coding. Codes were developed and modified through the coding process. Data were coded separately, and then developed a thematic framework with all data from FGDs and KIIs. When analyzing data from focus groups, the focus was mainly on a group as a whole rather than individual perspectives. Two research assistants and the principal researcher read the transcripts, coded the data and then compared the generated codes for similarities. Subsequently, we developed a codebook. Once saturation was reached, themes were identified, reviewed, and modified. Then we put together all data relevant to each theme and defined them. The identified themes were used in the write-up. In the study findings, we only used titles and number codes to present responses from participants. We did not include personal identifiers such as names and contact information. The data was managed in Nvivo version 12.0.

## Results

There were 48 participants in the FGDs and 19 Key informants leading to a total of 67 participants for the whole study. Mothers’ age ranged from 17 to 38 years. Most of them were employed and had secondary education. The health workers were 23–55 years, with the majority having a bachelor’s degree and only one with a post-graduate degree. Grandmothers were 46–63 years, employed and most had attained a primary level of education. All fathers were employed, had acquired secondary education, and their ages ranged from 28 to 46 years. The demographic characteristics are presented in Table 1.

**Table 1:** Characteristics of participants

Groups		No. of participants	Mean age in years (range)	Mean years of Education (range)	Mean No. of children (range)	No. employed
Focus groups	Pregnant mothers	12	26.33 (17–35)	10.83 (7–16)	1.83 (1–4)	8
	fathers	12	34.58 (28–46)	10.75 (4–17)	3 (1–6)	12
	grandmothers	12	52.42 (46–63)	7.67 (3–16)	3.5 (1–8)	9
	lactating mothers	12	27.75 (20–38)	11.58 (2–16)	2.08 (1–4)	11
Key informant interviews	nurses	4	37.25 (30–50)	15.5 (13–17)	2.25 (1–3)	4
	paediatricians	2	42 (38–46)	19.5 (18–21)	2	2
	midwives	4	38.5 (23–55)	15 (13–17)	2 (0–4)	4
	Mothers ever used DBM	3	31 (24–37)	16.33 (16–17)	2.33 (1–4)	3
	Mothers of preterm babies	6	28.5 (17–36)	10.67 (5–16)	2.33 (1–5)	4

Perceptions towards the use of donated breast milk were identified through two major themes presented in Table 2. The perceptions were categorized between positive and negative perceptions towards donated breast milk. Positive perceptions: This theme describes the participant’s view and interpretation of using donated breast milk for infant feeding as something good. The positive perception included the nutritional value of breast milk, donated breast milk just like blood donation, an opportunity for babies who would otherwise not get breast milk, and the opportunity to avoid feeding babies formula or cow milk. Negative perceptions: This theme describes participants’ beliefs or views that predict donated breast milk to be unfit or not suitable for infant feeding. The negative perceptions were about inheriting the genes and bad traits of the donor, the feeling that donated breast milk is disgusting, safety and quality issues, affecting the bond between mother and child as well as the fear of high prices. These can be substantiated as follow.

**Table 2:** Participants’ perceptions on the use of donated breast milk

*Main theme*	*Sub theme*	*Codes*	*Quotations*
Positive perceptions	Nutritious	• Its human milk • Has all nutrients the baby needs	• “Donated milk is from humans and the one receiving it is a human. It will definitely have nutrients that are better than tinned milk.” (Participant 4, FGD fathers, Nsambya hospital). • Breast milk contains everything, the white blood cells that prevent a baby from acquiring infections. It can easily be digested. You won’t have a baby with diarrhoea because of it. Baby won’t vomit it, ok, it’s just healthy. It’s part of the human body. The way you see its importance I would recommend it to my own child. (KII, Nurse, Nsambya Hospital). • “Breast milk can’t be substituted in terms of nutrients. It is what God created and is the best for babies.” (KII, Mother of premature ever used DBM, Nsambya hospital).
		• Breastfed babies are healthy	• “The truth is, milk that comes from mothers is the kind of milk where a baby is in good health and fully cared for. Whenever a baby is not given this breast milk and you prefer going to work and the baby doesn’t breastfeed that is where we find malnourished babies because you have not cared enough for this baby.” (Participant 3, FGD grandmothers, Naguru hospital).
		• The right balance of nutrients. • Properly measured	• DBM has all the nutrients. It’s not that you’re going to give NAN, sometimes those mothers that give NAN, they don’t know the right mls to give, and they don’t know how to mix it. Sometimes if you leave the baby with a maid, they give the baby, for example they haven’t boiled the water very well, and the baby will get other things that lead to complications. But if the breast milk is got, at least I know it has everything in its correct amount. (KII, Midwife, Nsambya Hospital). • “Tined milk may not have directions from the doctor yet this one definitely has directions from the doctor and is properly measured. But for tined milk you can buy and just mix without knowing the right measures to us.” (Participant 1, FGD fathers, Naguru hospital).
	Help babies that would not get breast milk	• Help babies & mothers in need • Baby gets a taste of breast milk	• “It is going to help mothers who don’t have breast milk and also children who have lost their mothers, they can get the taste of breast milk because it is the best.” (KII, Midwife, Naguru hospital). • “It helps, some parents immediately after giving birth she dies. It helps the baby to at least get breast milk.” (Participant 2, FGD Lactating mothers, Naguru Hospital).
	Opportunity to avoid feeding babies formula or cow milk	• Saved from cow’s milk • Less use of formula milk • Go for human than animal milk	• “Me, I have no problem with it because the baby receives milk that is right, natural and saved from using cow’s milk.” (Participant 2, FGD Grandmothers, Naguru hospital). • “If we can get a breast milk bank, the formula would lose the market, no worries of a baby over feeding or mixing water, worrying of adding too much water or too much formula. It is just standard.” (KII, Mother of premature ever used DBM, Nsambya hospital). • “Hmmm, instead of me buying cow’s milk when there’s human milk, I rather go for human milk. Not milk from animals.” (Participant 2, FGD fathers, Nsambya hospital).
	Same as blood donation	• Like donating blood to someone • Blood transfusion • Need of blood	• “The thing is ok. It’s like donating blood to someone.” (KII, Nurse, Nsambya Hospital). • “I think it’s good because, just like how we can use blood transfusion and you are trying to save a life and for preterm they require to get breast milk, it is the best option for them.” (KII, paediatrician, Nsambya hospital). • Now, it’s like bringing a patient to the hospital, and they tell you this person needs blood. So if this milk is also prepared properly and carefully, and there is an emergency, there is no problem as long as it helps improve the health of this person. (Participant 5, FGD fathers, Nsambya hospital).
Negative perceptions	Disgusting	• Feel disgusted	• “Another woman’s milk is disgusting.” (Participant 6, FGD pregnant mothers, Nsambya hospital). • “Hmmm, some mothers will take it like, forgive me for using this word “ekyenyinyarwa” (disgusting). Because this milk is mixed from different donors.” (Participant 3, FGD fathers, Naguru hospital). • “I even feel disgusted about it. I like when it is mother to baby not in a bottle. Some mothers are unhygienic they are disgusting (benyinyaza).” (KII, midwives, Naguru hospital).
	Inheriting genes & negative traits of the donor	• Mixed a lot of different blood • Transfer bad traits/ character/ lifestyle • Habits of theft	• “Hmmm, this milk isn’t bad, but it has mixed a lot of different blood.” (Participant 3, FGD fathers, Naguru hospital). • The problem that comes with the mixing of blood from different people, is what I look at as a problem. For instance, you may not be cannibalistic, but these different kinds of blood that a child has breastfed on! That is what am worried about because it is donated by people of different blood. (Participant 3, FGD grandmothers, Naguru hospital). • Every clan has its own stuff. You don’t know this person’s blood group. You don’t know whether this clan behaves like this, whether they have sickle cells which make you refuse this milk. On this side they are thieves, on the other, they are cannibals, and you wouldn’t want your baby to become a thief just because she breastfeeds on another person’s breast milk. (KII, mother of preterm, Naguru Hospital).
		• Mixing/transfer genes • Behave like the donor	• “…genes might be transmitted through donor milk like habits of theft.” (KII, midwives, Naguru hospital). • “Genes are very important and those traits cannot be ruled out by the health worker. The child might end up behaving like the donor or having habits of a donor.” (Participant 2, FGD grandmothers, Nsambya hospital).
		• Taboo to feed baby another woman’s breast milk	• “There those who feel its taboo for you to give another woman’s breast milk to your baby and will obviously not accept.” (KII, paediatrician, Naguru hospital).
		• Change child’s DNA	• “If they take the baby for DNA test won’t the baby be connected to the donor. What if she steals my child and they check her blood, won’t they find when she is connected to the donor.” (Participant 4, FGD Lactating mothers, Naguru hospital).
	Safety and quality concerns towards DBM	• Concern for allergies	• “It might not connect with the baby, and he or she reacts by vomiting or constipation.” (Participant 1, FGD lactating mothers, Naguru hospital).
		• Risk of infections & diseases	• “The donor is available but has either HIV or hepatitis or syphilis, any kind of disease.” (KII, Mother of premature ever used DBM, Nsambya hospital). • “infections that are acquired such as HIV.” (KII, Nurse, Nsambya hospital).
		• Risk of feeding baby contaminated milk	• “What might scare me are the preservatives and other things added which are different from the real milk.” (Participant 4, FGD fathers, Naguru hospital). • Naturally we know that breast milk will eventually go bad if you keep it. But now when you preserve it for a long time, it means there are some preservatives added which in a long run won’t be good for the baby health-wise. (Participant 3, FGD fathers, Nsambya hospital). • “Contamination, external in terms of handling during expression and storage.” (KII, Mother of premature ever used DBM, Nsambya hospital).
		• Not stored appropriately • Over stayed/ expired milk • Mix old & new batch	• With donated breast milk, just a small error can cause my child to have different kind of diseases because at times it can be stored poorly. The baby can be in an emergency, you might find the milk was checked properly and donor screened well before storage, but then it takes long in the fridge or expires. By the time they give it to the child you find it’s not of the right standard. During that time when a child is in need, we might not have that time to recheck or ascertain the standard of that milk. So you give this milk to the baby, hmmm, “kumbe” (yet), anything can happen. (Participant 1, FGD Lactating mothers, Nsambya Hospital). • “You might find it wasn’t stored appropriately or they mixed old with new batch yet some has over stayed. So a lot can happen.” (Participant 2, FGD Lactating mothers, Nsambya Hospital).
		• Unhygienic • Poor hygiene • Transportation condition	• “Unhygienic person to feed my baby, may contaminate it and infect my baby” (Participant 1, FGD pregnant mothers, Nsambya hospital). • “Hygiene of the donor and also the transportation condition from the donor to the bank.” (Participant 2, FGD fathers, Nsambya hospital). • “Hygiene, how the milk is handled. Do they give all the tips on how to handle that milk?” (Participant 1, FGD Lactating mothers, Naguru Hospital).
		• Feeding of the donor • Cannot reach the standard for feeding babies.	• “Can’t know the feeding of the donor.” (Participant 5, FGD fathers, Nsambya hospital). • I can’t trust donated breast milk to reach the standard to which I would feed my baby. And also because I don’t keep it myself. You see even cow’s milk you have to first check if it’s not yet spoilt or dairy milk. But with donated breast milk remember even people who bring it or administer it know a lot. You can’t just tell them you want to first check if the milk is fine. All in all, hmmmm, I wouldn’t trust donated breast milk very well. (Participant 2, FGD lactating mothers, Nsambya hospital).
		• Distrust in the health condition of the donor • Distrust in screening procedures & health workers • False test results	• “If she is not screened when she has diseases that might affect the baby.” (Participant 3, FGD Lactating mothers, Naguru Hospital). • “You might find the donor has HIV but consistently taking drugs and tests negative for HIV test.” (Participant 1, FGD lactating mothers, Nsambya hospital). • “…telling the truth, I can have that doubt that the doctors might not tell the truth. What if someone bribes them or they screen and results are different from the reality.” (Participant 2, FGD lactating mothers, Naguru hospital).
	Fear it could be expensive	• Free/ sold • Expensive • Hard to access the milk	• Will it be for free? Hmm!! I don’t think it will be for free and if a mother can’t sustain herself to eat well and get breast milk, I don’t think she can buy that milk. Unless it’s going to be for free but if it’s for buying it will be difficult. (KII, Nurse, Naguru hospital). • That practice! Hmmmm, it’s not bad but though expensive. If you find someone who even let me say, fights hard to get at least two thousand shillings; and your telling this person we need only that milk. I don’t think a donor is going to give it to us free of charge, not the hospital. Because the two hospitals have so far heard where it is, I don’t think they are very cheap. Me myself I don’t think they are very cheap because I think you have to pay the donor. So there am not yet sure whether you pay the donor or the institution and how. Because, and how many mls are you going to pay per donor? Are you going to pay per day or a contract of six months? (KII, Midwife, Nsambya hospital). • “Me, I can only fear that it could be expensive. We don’t know whether it will be for free or sold.” (Participant 3, FGD grandmothers, Nsambya hospital). • “…a lot of logistic hurdles might be needed to access the milk…” (KII, paediatrician, Naguru hospital).
	Affect mother to child bond	• Miss bonding • Mother’s love goes away • Neglect of babies	• “That feeling stays in me that my baby will get love distant from mine, and she misses that bond between me and her as her mother.” (Participant 1, FGD lactating mothers, Naguru hospital). • “It has a negative effect on our mothers, they will now neglect…doesn’t mind because there is that milk.” (Participant 6, FGD fathers, Naguru hospital).

### Positive perceptions

The majority of the study participants in both FGDs and KIIs perceived donated breast milk as the same as the biological mother’s milk. Most participants believed *“donated breast milk is from humans and will definitely have better nutrients.” (Participant 4, FGD fathers, Nsambya hospital)* than other manufactured feeding options. All mothers of preterm babies that had previously been exposed to the practice or idea of donated breast milk were receptive. Mothers of preterm babies strongly believed breast milk to be superior in nutrients required by preterm babies. A mother of a preterm baby that had used donated breast milk before mentioned, *“It is a very good idea; breast milk can’t be substituted in terms of nutrients. It is what God created and is the best for preterm babies.” (KII, mother of premature ever used DBM*, *Nsambya hospital).* From FGDs with fathers and grandmothers, they emphasized that donated breast milk is already in correct nutritional measurements, which is not the case with the other baby foods.

*Breast milk has vitamins and proteins. It has everything a baby needs and in the right quantity, made by God. It is not like those formulas that are man-made. It is possible that some nutrients are not well balanced. (Participant 3, FGD grandmothers, Nsambya hospital).*

*Breast milk contains everything, the white blood cells that prevent a baby from acquiring infections. It can easily be digested, and healthy, and it is part of the human body. The way you see its importance, I would recommend it to my child. (KII, Nurse, Nsambya hospital).*

Participants regarded donated breast milk as a measure to help babies who don’t have access to milk from their mothers. Some participants highlighted that donated milk would be a good initiative, especially for vulnerable babies and mothers in critical conditions or emergencies, while others die in labour/during childbirth. *“It helps, some parents immediately after giving birth she dies.” (Participant 2, FGD lactating mothers, Naguru hospital).* Some health workers pointed out that sometimes mothers give birth to more than one or two babies. Therefore, such mothers may not have enough milk to satisfy all the babies. They believed that such an arrangement of donated breast milk would be helpful as all the babies would benefit from using it, as opposed to other alternatives that are not as nutritious as breast milk. They believed such mothers would gladly embrace donated breast milk as an option.

Most of the health workers and mothers of premature babies supported the idea of donated breast milk because they had seen children suffer in the absence of their mothers. As put by a mother of a preterm baby; “*this would be good because parents can be desperately looking for this milk and are readily in need. So I would just recommend that it’s a good initiative for preterm parents to be able to get it.” (KII, mother of preterm, Nsambya hospital).* The mothers of premature babies perceived it as a long-awaited initiative that could reduce their stress. Some of the mothers had lived experiences whereby at one moment, they needed to look for breast milk or saw others in similar situations. Health workers noted that having donated breast milk would ease their work when recommending the appropriate baby feeds to the mothers. *“…. there are people who don’t have breast milk and we have been telling them to buy cow and gate milk, NAN, but donated breast milk would be another possibility.” (KII, Midwife, Naguru hospital).*

Furthermore, health workers and fathers regarded donated breast milk to be similar to blood donation. As put by one of the *Paediatricians; “It’s just like how we can use blood transfusion, and you are trying to save a life.” (KII, Paediatrician, Nsambya hospital).* Most of the participants perceived the use of donated breast milk as a lifesaving intervention. Fathers believed that donated milk would only be beneficial and acceptable if used when there is no alternative, especially in the absence of a mother or in a life-threatening condition.

*Now, it’s like bringing a patient to the hospital, and they tell you this person needs blood. So if this milk is also prepared properly and carefully, and there is an emergency, there is no problem. (Participant 5, FGD fathers, Nsambya hospital).*

Furthermore, participants in focus groups and KIIs perceived the use of donated breast milk as an opportunity to avoid feeding babies formula or cow milk. They felt glad and relieved that their babies would feed donated breast milk than other forms of breast milk alternatives. This perception was because they believed donated breast milk would be more nutritious and well-balanced vis-a-vis the other alternatives. As noted by one of the fathers: *“Hmmm, instead of me buying cow’s milk when there’s human milk, I rather go for human milk. Not milk from animals.” (Participant 2, FGD fathers, Nsambya hospital).* One of the mothers of premature babies perceived that having the breast milk bank would reduce the use of formula milk and worries about preparation procedures. *“If we can get a breast milk bank, the formula would lose the market, no worries of a baby over-feeding or mixing water, worrying about adding too much water or too much formula. It is just standard.” (KII, mother of preterm, Nsambya hospital).* On the other hand, fathers and grandmothers believed establishing the human milk bank would increase breast milk consumption and reduce malnutrition. Participants highlighted that some mothers work and others do not like to breastfeed today. Hence, such mothers would use donated milk as an alternative. As put by the fathers:

*It’s not bad, but rather good considering today’s new generation. You may find someone who doesn’t want to breastfeed, and the baby gets malnourished. Yet she would buy and feed the baby. (Participant 3, FGD fathers, Naguru hospital).*

### Negative perceptions

Some health workers, lactating and pregnant mothers perceived the use of donated breast milk as completely unacceptable. Two of the four midwives and one of the nurses clearly stated that donated breast milk is disgusting. They were mainly concerned about the processes involved in acquiring and preparing the milk. Some participants continued to say that they even fail to express their milk because of their phobia as well as the bad feeling they experience when they see their breast milk. “*Even mine I fail to express it and put it in a bottle, just looking at it disgusts me, hmmm, I can’t do it.” (KII, Midwife, Nsambya hospital).* For such people, it is highly doubtful that they could use donated breast milk or even support the idea. One of the lactating mothers stated; *“For me, I may feel disgusted for real. How can a baby breastfeed on milk from another person? My fellow woman!” (Participant 3, FGD lactating mothers, Naguru hospital).*

The myth of inheriting genes and negative traits of the donor through donated breast milk was highlighted in all focus group discussions and by most of the key informants. Two midwives, four nurses, one paediatrician and three mothers of premature babies pointed out gene and trait transfer as a great concern towards donated breast milk. *“Genes are very important and those traits cannot be ruled out by the health worker.” (Participant 2, FGD grandmothers, Nsambya hospital).* Most of the participants believed a baby should receive breast milk from a person related to him/her. Such participants perceived that when a baby takes donated milk, they would be exposed to rather different traits and behaviours that accrue to the donors. Therefore, a child who would be well-behaved may become badly behaved or have some funny behaviours that are not a characteristic of the family where he/she is born. *“Maybe the baby will start behaving like the one who breastfeeds her.” (KII, Nurse, Naguru hospital).* It was also emphasized in the focus groups that the transfer of genes and traits is a great concern among men. *“Men don’t want to mix their blood because of different characters in the families*.” (*Participant 6, FGD grandmothers, Naguru hospital).*

In addition, participants perceived donated breast milk use would prevent bonding between mother and child and encourage irresponsible mothers. These views emerged as a perception in some of the FGDs and KIIs. In one of the focus groups with the lactating mothers and interviews with two nurses, it was clear that they perceived that the baby would connect more with the donor and not the biological mother. Some mothers noted that they would feel insecure and jealous of the other mother who donates milk to the baby: *“That feeling stays in me that my baby will get love distant from mine, and she misses that bond between me and her as her mother.” (Participant 1, FGD lactating mothers, Naguru hospital).* The nurses emphasized the importance of bonding between mother and child during breastfeeding: *“There is that bonding between the mother and the baby when the mother is breastfeeding. So I think, me as one, those mothers would prefer breastfeeding their babies.” (KII, Nurse, Naguru hospital).*

On the other hand, fathers were worried that donated breast milk might encourage irresponsible mothers. As put by one of the fathers:

*It has a negative effect on our mothers. One, they will now neglect. You see, sometimes she is at work and recalls that I breastfeed, let me run and check on my baby. Now, she will sit and not mind because of that milk. (Participant 6, FGD fathers, Naguru hospital).*

Participants perceived that donated breast milk would not be safe and therefore liable to transmitting diseases to the young ones. The diseases in question ranged from allergies and also HIV/AIDS, and this was attributed to the process of acquiring, handling, and storage of donated breast milk. The participants continued to say that transmission of diseases through donated breast milk was especially possible if the donor to the baby was infected. This perception was aired by almost all the key informants and in all focus groups. Some participants highlighted that a donor might test negative for HIV, yet she still has the virus. Also, other infectious diseases like hepatitis and other sexually transmitted infection were cited. *“The donor is available but has either HIV or hepatitis or syphilis, any kind of disease.” (KII, mother of premature ever used DBM, Nsambya hospital).* Some of our participants were reluctant to use donated breast milk because the only available donors were perceived to be infected with any of the diseases mentioned. As put by one of the lactating mothers:

*You might find the donor has HIV but consistently takes drugs and tests negative for HIV test. The virus might be just hidden however much they are screened, you can’t tell because they are consistently on ARVs, and tests turn out negative. (Participant 1, FGD lactating mothers, Nsambya hospital).*

All participants who accepted the use of donated breast milk emphasized the need for thorough screening of the donor to ensure that she is safe and free from diseases transmitted through breast milk. As put by one of the mothers of premature babies: *“As long as the donor is well screened for all the diseases they say they have to and can be transferred through breast milk…” (KII, mother of preterm, Nsambya hospital).*

“Process of acquiring, handling, and storage of donated breast milk” also was perceived as a great safety concern among the participants. These concerns were mainly related to hygiene, distrust in health workers, transportation, collection, processing, and preservation procedures.

Some of the participants in FGDs and KIIs expressed distrust in the hygiene of the donated milk. One of the midwives and three of the mothers of premature babies were concerned with the risk of feeding babies contaminated milk because of poor hygiene and handling of the milk by the donor and health workers: *“Hygiene, the milk can get easily contaminated.” (KII, Midwife, Naguru hospital). “Hygiene of both the donor and those that are going to keep it.” (Participant 3, FGD lactating mothers, Naguru hospital).*

Discussions with the fathers, grandmothers, and lactating mothers revealed that they were concerned about the storage conditions and the shelf-life of the donated milk. Participants noted that some preservatives may be added to the donated milk and are not informed. Others highlighted that *“You might find it wasn’t stored appropriately, or they mixed old with the new batch. Yet, some have overstayed.” (Participant 1, FGD lactating mothers, Nsambya hospital).* Thus a perception that this kind of milk may not be safe for the babies that need it.

Furthermore, fathers and pregnant mothers were concerned about the practicalities. For example, *“The transportation condition from the donor to the bank.” (Participant 2, FGD fathers, Nsambya hospital);* if donation procedures are not done at the hospital. Some mothers were worried about the sensitivity and the need to train their caretakers on donated breast milk use. As put by one of the pregnant mothers *“…it may require first training the person you are going to leave that milk with. It needs your presence to give that milk…” (Participant 1, FGD pregnant mothers, Nsambya hospital).*

Furthermore, from the discussions with lactating mothers, they expressed doubt about the process of acquiring donated breast milk. Mothers highlighted that they lack trust in the efficiency of the process of breast milk banking. Particularly the screening procedures as well as health workers. Participants noted that there could be a possibility of false results since some doctors may not give it much attention. Others felt that, given that health workers are more knowledgeable, they would be scared to question them about the milk before giving it to their babies. As expressed in the following quotes:

*Also telling the truth. I can have that doubt that the doctors might not tell the truth. What if someone bribes them or they screen and the results are different from the reality? (Participant 2, FGD lactating mothers, Naguru hospital).*

*But with donated breast milk, remember even people who bring it or administer it know a lot. You cannot just tell them you want to check first if the milk is fine. (Participant 4, FGD lactating mothers, Nsambya hospital).*

Lactating mothers were concerned about “allergic reactions”. Some participants perceived that donated breast milk might cause allergies in their babies. As put by one of the lactating mothers: *“It might not connect with the baby, and he or she reacts.” (Participant 1, FGD lactating mothers, Naguru hospital).*

Also, participants perceived donated breast milk as being sub-standard. In one of the focus groups with lactating mothers, they stated that they do not trust the quality of donated breast milk to be as good as the food they would feed their babies: *“I can’t trust donated breast milk to reach the standard to which I would feed my baby.” (Participant 2, FGD lactating mothers, Nsambya hospital).* This perception was mainly related to the nutritional quality of the donated milk. Mothers and fathers highlighted that they *“can’t know the feeding of the donor, yet some women have poor feeding habits*.*” (Participant 5, FGD fathers, Nsambya hospital).* Hence, compared to the biological mother’s milk, donated milk could lack some nutrients the child needs to meet their developmental milestones.

The majority of the participants also perceived that donated breast milk would be very expensive to purchase, so only the rich would be able to afford it. This perception was because of the fact that its nutritional value is very high and well balanced. Given this quality, it would go for a higher price which would not easily be managed by all the people in need. Some of the midwives noted that they would only recommend it to those who can afford the service as they believed a lot of expenses are incurred in processing and acquiring the milk. Also, some participants were concerned about whether the donation of this milk would be free at the hospital. In as much as it would be donated freely, they were not sure whether the people who required it would get it free of charge, and therefore a feeling that it would be expensive.

*Will it be for free? Hmm!! I don’t think it will be for free and if a mother can’t sustain herself to eat well and get breast milk, I don’t think she can buy that milk. Unless if it’s going to be for free but if it’s for buying it will be difficult. There are those few who would want to, but then the majority! And those few who can they produce few babies, that one you know. Rich people who would opt for that produce few babies. (KII, Nurse, Naguru hospital).*

*That practice! Hmmmm, it’s not bad but though expensive. If you find someone who even let me say, fights hard to get at least two thousand shillings; and your telling this person we need only that milk. I don’t think a donor is going to give it to us free of charge, not the hospital. (KII, Midwife, Nsambya hospital).*

### Perceived strategies to address the negative perceptions

In all focus groups and interviews, participants suggested several strategies to address the negative perceptions towards the use of donated breast milk. Sensitization and health education was the first strategy mentioned by all participants. They highlighted a need for community training, sensitization through the media, health education programs at antenatal clinics, the use of champions, and educating health workers on human milk banking. Health workers felt a need to be well-informed about donated breast milk in case some people have misconceptions about it. They highlighted that there is a possibility that some health workers might also be negative about donated breast milk due to a lack of experience with it or breastfeeding.

*Mothers need to be educated. For example, during antenatal, they should include teachings on donated breast milk so that mothers who come for antenatal are educated. So, by the time they give birth and don’t have breast milk, it’s not a surprise. (Participant 2, FGD grandmothers, Nsambya hospital).*

*You teach us more about that. You know I can be a health worker, there is a mother who can accept but me as a health worker, I don’t like it. So, I won’t be able to tell the mother. But if l learnt more about it, I know I can be able to tell it to another person. (KII, Midwife, Nsambya hospital).*

Participants emphasized that the focus should be on the donors, the safety of the milk, the benefits and processes involved in human milk banking, and the repercussions of not using breast milk. Other suggestions included: screening the donor, having no financial attachment to donated milk, establishing the bank, using donated milk only for emergencies, men’s involvement, and standardization of the donated milk.

## Discussion

This study explored the perceptions of mothers, fathers, and health workers on donated breast milk use.

Study participants expressed positive perceptions such as donated breast milk helping babies who would miss out on mother’s milk, having nutrients comparable to biological mother’s milk, and similar to blood transfusion. However, some participants perceived using donated breast milk as negative due to the likelihood of disease transmission and passing on genes and bad traits from the donor to the baby. It is understood that people’s experiences and the world around them shape their perceptions. The positive perceptions in our study may be because some participants had been exposed to donated breast milk before or because health workers understand the significance of breast milk. A study in Limpopo Province, South Africa, reported that health workers know the value of donated breast milk and are familiar with human milk banking [27]. Similarly, Coutsoudis et al., 2011, clearly illustrated that people with prior experience and exposure to donated milk were more convinced of its value and efficacy [18]. These findings are consistent with that of our study.

The findings that donated breast milk has nutrients comparable to those found in mother’s milk and is better for babies than other forms of baby food alternatives agree with a cross-sectional study conducted among nurses and midwives in Tabriz, Iran [28]. The study reported that participants agreed that donated breast milk is more nutritious than infant formula [28]. Similarly, a qualitative study of caregivers and health workers in Eastern Uganda reported that participants perceived donated breast milk as beneficial compared to other forms of feeding [29]. Correspondingly, a study conducted in south-east Nigeria demonstrated that 98% of mothers believed breast milk is the best feed for infants, because it is full of nutrients that are superior to those in other foods [14]. In addition, the finding on donated breast milk providing an opportunity to avoid formula or cow milk is in line with that of the UK study [30]. The study reported that women were relieved and grateful for not having formula milk as part of their feeding plan [30]. Similar findings were reported in Kenya [31]. Donated breast milk is considered the first alternative in the absence of the biological mother’s milk [12–14].

The finding on participants perceiving donated breast milk use as an opportunity for babies who do not have their mother’s milk differs from other studies. Studies report sympathy and generosity as to why most mothers would donate breast milk than use it [32–34]. This could be attributed to the study population used in this study. Some mothers had been through a similar experience, where they could see their babies struggling and needed donated breast milk. Others had vulnerable babies who were born prematurely, and health workers had seen mothers going through the struggle. In the UK, a study conducted among parents who had received donated breast milk reported that participants perceived it as beneficial to both the mother and baby and were relieved to know their babies had the opportunity to receive breast milk [30]. The findings from this study were in agreement with those from our study.

Furthermore, the study revealed that participants regarded donated breast milk use the same as blood donation. This finding implies that knowledge of the benefits of blood donation would foster the donation and use of breast milk. Contrary to this finding, a South African study demonstrated that irrespective of some participants relating donated breast milk to blood transfusion, mothers were more comfortable with blood donation than using donated breast milk [18].

Despite the positive perceptions towards the use of donated milk, most of the participants were reluctant towards the idea of using another woman’s breast milk. The major reasons given are not any different from what has been reported in other studies. The possibility of transmitting diseases, hygiene and inheriting bad traits from the donor through donated breast milk. The perception around the safety of donated breast milk is in line with the findings of a qualitative study conducted in eastern Uganda, which demonstrated that participants were worried that the use of donated breast milk would transmit infectious diseases such as HIV and non-communicable diseases [29]. Kimani-Murage et al. (2019) found out in their study that mothers in Kenya did not want to feed their babies donated breast milk because they believed it could potentially harm their children by transmitting HIV and other diseases [31] Studies in eastern Ethiopia, Benin and New York have reported similar findings [11, 35, 36]. Like this study, several other studies have reported that participants would only use the donated breast milk if assured that the donor is appropriately screened and the milk is deemed safe [14, 18, 31, 37, 38]. In addition, studies in south-east Nigeria and Turkey reported that participants perceived donated breast milk as unhygienic [14, 39]. This finding was mainly related to how donated breast milk is acquired, stored and handled. A qualitative study conducted at Mbale Regional Referral Hospital, Uganda, found that the perceived hygiene of the donor and during the process of acquiring donated breast milk was important among caregivers [29]. Similarly, a mixed-methods study conducted in Nairobi, Kenya, that investigated the perceptions on donating and using donated breast milk reported hygiene as a concern raised among mothers [31]. In the study of Varer Akpinar et al., women in Turkey mentioned that they didn’t feel comfortable feeding their babies breast milk from another woman because they perceived it as unclean [40]. Similarly, in Ethiopia, distrust in hygiene when collecting donated breast milk was one of the reasons why mothers wouldn’t feed their babies on donated breast milk [35].

Furthermore, the perception of a baby inheriting genes and bad traits from the donor through donated breast milk agrees with a study in South Africa that investigated stakeholder attitudes towards donated breast milk [41]. The study reported that fear of the transfer of personal traits was a concern raised among the participants [41]. In addition, the present study revealed that men were mainly worried about mixing blood with regard to using donated milk. There is limited literature on how men perceive the use of donated breast milk. However, studies in South Australia, India and south-east Nigeria have reported that men influence women’s decisions on the use of donated breast milk [14, 37, 38]. Similar to our study findings, a study conducted in Kenya reported that participants supposed that donated breast milk use would negatively affect the emotional bond between a mother and their child [31]. A study in South Africa reported similar findings [41]. Contrary to these findings, Brown and Shenker found that donated breast milk strengthens the bond between a mother and her child since it reduces the pressure on them to produce sufficient breast milk [30]. These findings emphasize the misconceptions and social-cultural beliefs surrounding donated breast milk use.

On the other hand, some mothers and health workers in the study perceived donated breast milk as disgusting. This perception was more articulated in the pregnant mother’s focus groups. These views could be because donated breast milk is a body fluid, and hence there is a certain degree of sensitivity towards its use. This finding has been reported before in other studies [18].

In addition, the perception that donated breast milk is expensive has been found in other studies. In South Australia, mothers perceived donated breast milk as expensive and emphasized a need for no cost attached to it or to be charged less than formula milk [38]. This finding could be due to the social-economic status of the large majority in Uganda.

This study found that participants had negative perceptions of donated breast milk because of misconceptions about its safety and their social-cultural beliefs. However, exposure to donated breast milk and breastfeeding experience contributed to the positive perceptions. These findings imply that there is a need for sensitization and education programs focusing on the acquisition procedures, handling and storage of donated breast milk, as well as the importance of donated breast milk. In addition, participants expressed a need to educate health workers on donated breast milk. Health workers influence how the public perceives donated breast milk [21, 27, 31]. A hospital-based study in eastern Uganda revealed that health workers encouraged mothers to use donated breast milk [29]. These findings suggest a need to design targeted education programs when addressing perceptions towards donated breast milk.

The study findings offer new perspectives into the likely negative and positive perceptions about donated breast milk use in central Uganda. Therefore, future studies should examine how to address these perceptions at different levels.

### Limitations

Study limitations include conducting the study among some participants who had limited information about donated breast milk. However, our findings provide important insights into potential negative and positive perceptions towards the use of donated breast milk among mothers, fathers and health workers in low-income countries. We could not find any theoretical framework from similar studies in the literature to inform our study. Therefore, we only described participants’ perceptions from the discussions and interviews. In addition, the findings were specific to the urban and hospital context. Hence, there could be bias against donated breast milk since people from the community and rural areas may have different perceptions.

## Conclusion

In summary, participants had positive perceptions about donated breast milk but were concerned about the potential side effects. Health workers should take extra precautions to ensure that donated breast milk is safe to reassure the participants. The development of appropriate information and communication programs to sensitize the public about the benefits of donated breast milk will improve the uptake. These programs can be utilized most especially by health workers and the ministry of health to promote breastfeeding. Further research should focus on understanding the social-cultural beliefs regarding donated breast milk.

## Data Availability

The datasets used and/or analyzed during the current study are available from the corresponding author upon reasonable request.

## Abbreviations

DBM: Donated Breast Milk
DBMB: Donated Breast Milk Banking
FGD: Focus Group Discussion
HMB: Human Milk Banking
KII: Key Informant Interviews
NICU: Neonatal Intensive Care Unit
WHO: World Health Organisation
PD: Paediatrician
